# Ethnic Variability in Body Size, Proportions and Composition in Children Aged 5 to 11 Years: Is Ethnic-Specific Calibration of Bioelectrical Impedance Required?

**DOI:** 10.1371/journal.pone.0113883

**Published:** 2014-12-05

**Authors:** Simon Lee, Vassiliki Bountziouka, Sooky Lum, Janet Stocks, Rachel Bonner, Mitesh Naik, Helen Fothergill, Jonathan C. K. Wells

**Affiliations:** 1 Respiratory, Critical Care and Anaesthesia Section, UCL Institute of Child Health, London, United Kingdom; 2 Postgraduate medical education, Guy's and St Thomas' NHS Foundation Trust, London, United Kingdom; 3 General Practice Vocational Training Scheme, North Devon District Hospital, Barnstaple, United Kingdom; 4 Childhood Nutrition Research Centre, UCL Institute of Child Health, London, United Kingdom; Faculty of Biology, Spain

## Abstract

**Background:**

Bioelectrical Impedance Analysis (BIA) has the potential to be used widely as a method of assessing body fatness and composition, both in clinical and community settings. BIA provides bioelectrical properties, such as whole-body impedance which ideally needs to be calibrated against a gold-standard method in order to provide accurate estimates of fat-free mass. UK studies in older children and adolescents have shown that, when used in multi-ethnic populations, calibration equations need to include ethnic-specific terms, but whether this holds true for younger children remains to be elucidated. The aims of this study were to examine ethnic differences in body size, proportions and composition in children aged 5 to 11 years, and to establish the extent to which such differences could influence BIA calibration.

**Methods:**

In a multi-ethnic population of 2171 London primary school-children (47% boys; 34% *White*, 29% *Black African/Caribbean*, 25% *South Asian*, 12% *Other*) detailed anthropometric measurements were performed and ethnic differences in body size and proportion were assessed. Ethnic differences in fat-free mass, derived by deuterium dilution, were further evaluated in a subsample of the population (n = 698). Multiple linear regression models were used to calibrate BIA against deuterium dilution.

**Results:**

In children <11 years of age, *Black African/Caribbean* children were significantly taller, heavier and had larger body size than children of other ethnicities. They also had larger waist and limb girths and relatively longer legs. Despite these differences, ethnic-specific terms did not contribute significantly to the BIA calibration equation (Fat-free mass = 1.12+0.71*(height^2^/impedance)+0.18*weight).

**Conclusion:**

Although clear ethnic differences in body size, proportions and composition were evident in this population of young children aged 5 to 11 years, an ethnic-specific BIA calibration equation was not required.

## Introduction

Information on children's body composition is increasingly used in research studies [1]–[3]. Although such information could improve understanding of the magnitude of the obesity epidemic, and could also improve the diagnosis and management of diverse paediatric diseases, such data are rarely obtained for use in public health monitoring or clinical practice [4].

The most commonly used measure of body fatness at the population level is body mass index (BMI) [5]. Large scale studies, such as the Millennium Cohort Study, frequently use BMI as a proxy for body fatness [6], [7]. However, there are two major challenges with interpreting BMI. Firstly, it provides no information on the relative proportions of fat mass (FM) and fat-free mass (FFM) [8]. Two children of the same age and sex with the same BMI can have a twofold range of FM [9]. Secondly, the relationship between BMI and body fatness is affected by ethnicity. For a given BMI, *South Asian* children have more body fat [10], [11] and *Black African/Caribbean* children less, when compared with *White* children [11]. To overcome these limitations, other more accurate methods of measuring body fatness are available, such as densitometry, magnetic resonance imaging, isotope dilution and multi-component model [12]. However, these specialized techniques are time-consuming, expensive and tend not to be widely available.

Bioelectrical impedance analysis (BIA), which measures the impedance of the body to a small electrical current, represents an inexpensive, non-invasive and portable approach. This technique requires an equation to convert raw data on bioelectric properties and anthropometry to final body composition values (Total Body Water (TBW), or FFM). Some equations, which follow the classic approach of treating the body as a cylinder, predict body composition from the ‘impedance index’ (height^2^/impedance [HT^2^/Z]). Other equations which incorporate height and impedance (Z) separately have also been used. These BIA calibration equations are population-specific [13]–[15], such that BIA will not necessarily produce accurate results unless the equation is carefully chosen on the basis of the population characteristics (e.g. age, sex, health condition) [16], [17].

Previous studies have demonstrated that different ethnic groups also show different coefficients in the relationships between bioelectrical data and body composition [18]–[20], indicating that ethnic-specific BIA equations may be required. Ethnicity is a complex construct, incorporating both cultural phenomena (e.g. identity, customs) [21] and biological responses to long-term contrasts in living conditions [22]. The most likely explanation for ethnic variability in BIA equations is the well-established differences in physique and body proportions that are known to exist between ethnic groups [23], [24]. Given that impedance is affected by the cross-sectional area and the length of the body's conducting segments [25], it is possible that ethnic difference in body size and proportions, such as limb cross-sectional areas and the ratio of trunk to limb lengths, could contribute to the ethnic-specific relationship between bioelectrical data and body composition.

The aim of this study was to produce a calibration equation which maximises prediction accuracy within as well as across ethnic groups, using data from the Size and Lung function In Children (SLIC) study based in London, UK (http://www.ucl.ac.uk/slic) [26]. We first examined whether there were ethnic differences in body size, proportion and composition, and then calibrated BIA against deuterium dilution analysis, testing whether ethnic-specific terms were required.

## Methods

### Subjects

The SLIC study is an epidemiological study conducted in London primary schools, aiming to explore ethnic differences in lung function in a multi-ethnic population of children. [26]. This study was granted ethical approval by the London-Hampstead Research Ethics Committee (REC 10/H0720/53). All children with written informed parental consent were eligible to participate. Between 2011 and 2013, 2171 children aged 5–11 were assessed. Parents classified their children into one of the four broad ethnic groups: “*White*” which included white European, Hispanic or Latino and Middle Eastern children; “*Black African/Caribbean*” which included Black African and Black Caribbean children; “*South Asian*” which included Indian, Pakistani, Sri Lankan and Bangladeshi children; and “*Other*” which included any other ethnicities such as Chinese or Filipino children, as well as children of mixed ethnic origins (e.g. *South Asian*/*White*). Standing height and weight were measured in all children.

#### Comparison of body size and proportion

Detailed anthropometric measurements including weight, standing and sitting height, and circumferences of the mid-upper arm (MUAC), waist and calf were performed in all children during the first year of the SLIC study, using established protocols (see Text S1 for details). This population is referred to as the ‘body size population’.

#### BIA calibration

A sub-sample of children in the SLIC study (n = 605), representing an even distribution of BMI and age within each ethnic group, participated in the BIA calibration study. Arm-leg BIA was performed using the Tanita BC418 (with whole-body values for impedance (Z in Ω) as the outcome) and was calibrated against deuterium dilution analysis (described below). These data were supplemented by additional data collected using an identical protocol, in 5- to 11-year-old children (n = 93) studied at Great Ormond Street Hospital [27]. This combined population is referred to as the ‘BIA calibration population’. Detailed information on how these two populations were derived is shown in Figure S1.

#### Deuterium dilution analysis

Deuterium dilution was undertaken to measure TBW, from which FFM was estimated assuming constant hydration of fat-free tissue. Deuterium oxide dosages were prepared using 99.8% purity deuterium oxide (CK Gas Product Ltd., Ibstock, UK) and were weighed using scales with accuracy to 0.01 grams. Each participant in the calibration study was given an oral dose of ∼0.05 g of deuterium oxide per kg body weight. Saliva samples were collected using cotton wool swabs prior to the dose and at least 4 hours after the administration of the deuterium drink. Children avoided food and drink 30 minutes before providing a saliva sample. Fluid consumption between the deuterium dose and the second saliva sample was documented. Saliva samples (pre and post dose) and dose sample were analysed for isotopic enrichment by Iso-Analytical Ltd. (Sandbach, UK) and at the Institute of Child Health using the equilibration method [28] and continuous flow isotope ratio mass spectrometry. For calculating TBW, it was assumed that deuterium dilution space overestimated TBW by a factor of 1.044 [29]. Correction was made for dilution of the deuterium dose by fluid consumption during the 4-hour equilibration period. FFM was calculated from TBW using age- and sex-specific values for hydration of lean tissue [30]. There is no evidence that ethnic groups differ in their FFM hydration in children [30].

### Statistical analysis

#### Anthropometric comparison

To assess ethnic differences in body size and proportions and compare changes across the age-range, children in the body size population were grouped into 7 age groups (5 to <6; 6 to <7; 7 to <8; 8 to <9; 9 to <10, 10 to <11 years and 11 to <12 years) and separated into boys and girls. Measurements of weight, height and BMI were expressed as z-scores to adjust for sex and age using the British 1990 reference [31]. Mean anthropometric measurements and their standard errors were calculated for each ethnicity in each age group.

#### Body composition data by deuterium dilution

To graphically present the relative amount of FM and FFM of children in the BIA calibration population, a Hattori body composition chart was used [32], [33]. Hattori charts illustrate four pieces of information: FFM index (FFMI) which was calculated as FFM/height^2^; FM index (FMI) which was calculated as (weight-FFM)/height^2^; BMI; and % fat. To assist interpretation, a regression line for the relationship between FMI and FFMI in each ethnic group was plotted on the Hattori graph. One-way ANOVA with Bonferroni comparison was used to compare the means of FMI and FFMI between ethnic groups.

#### Calibration of BIA against deuterium dilution analysis

Nested linear regression models were used to assess whether height, weight, sex and ethnicity were related to FFM calculated from the deuterium dilution. Two groups of regression models were analysed: group A contained models which derive equations for estimating FFM by fitting HT^2^/Z (the classic approach), while group B contained models which derive equations by fitting height and Z independently. In addition to the proportion of variance explained (R^2^) and the standard error of the estimate (SEE), the Bayesian information criterion (BIC), which is based on the log likelihood with a penalty based on number of parameters in the model, was used for model comparison. The models of better fit should provide high R^2^,low SEE and low BIC values. The significance level was set at 0.05. The R program (v. 3.1.0) was used to perform the analyses [34].

**Figure 1:**
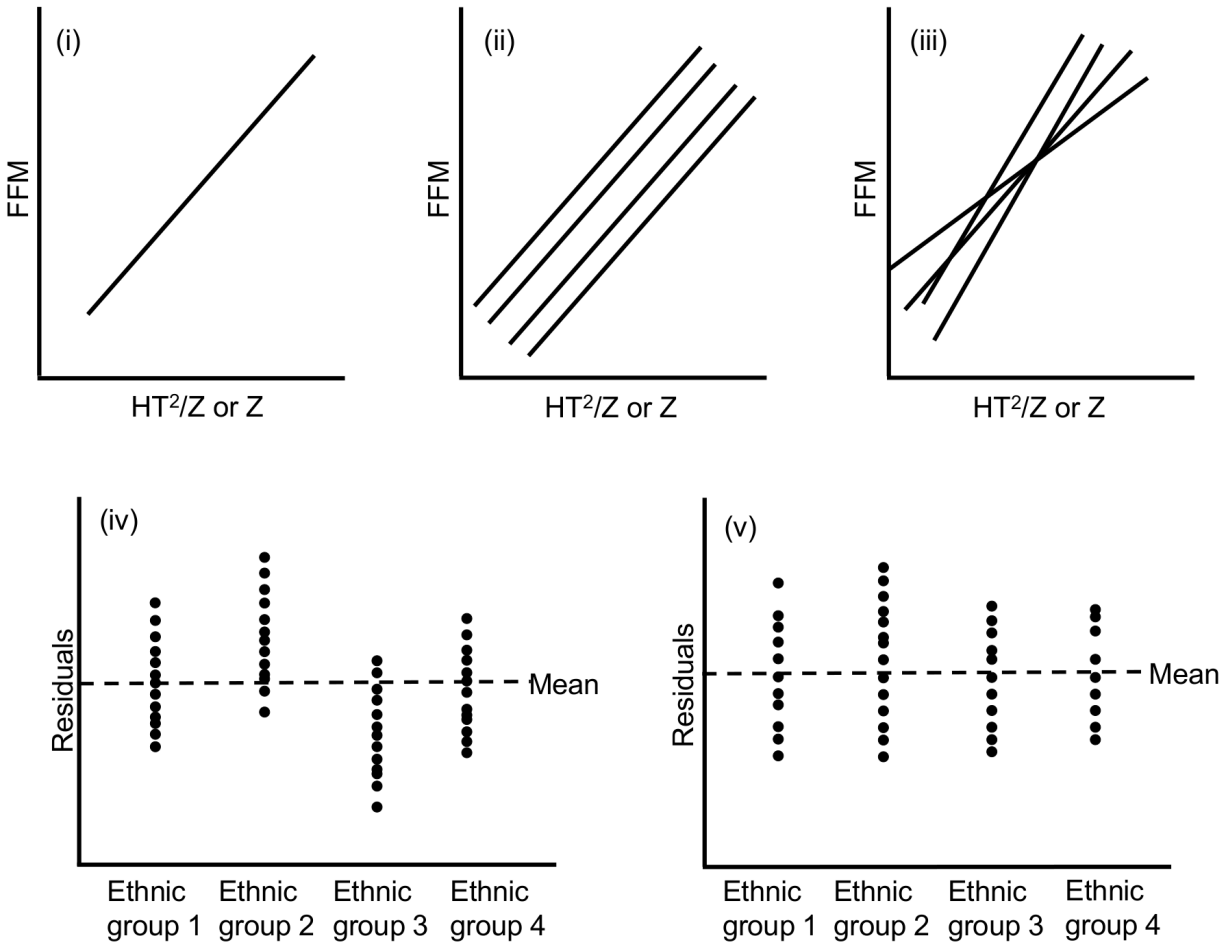
Conceptual illustration for deriving the ‘best’ BIA equation. Calibration equations aim to describe the relationship between fat-free mass (FFM) and height^2^/impedance (HT^2^/Z) or impedance (Z). Three types of calibration equations were investigated: (i) a generic equation with no ethnic terms; (ii) a simple ethnic-specific equation (same slope, different intercept); (iii) a complex ethnic-specific equation with ethnic-impedance interaction terms (different slopes, different intercepts). Model can have very similar average residuals but the distribution of residuals may vary across ethnic groups (iv). To avoid ethnic bias, models with even residual distribution (v) are preferred.

The conceptual illustration of the process used to derive the ‘best’ calibration equation is shown in Figure 1. The goal is to describe the relationship between FFM and HT^2^/Z or Z accurately and without any ethnic bias. The simplest approach incorporates no ethnic-specific terms (Figure 1i). The additive effect of ethnicity on FFM (i.e. constant difference of FFM across ethnic groups, providing identical slopes but different intercepts) was then tested. Next, the interactive effect between ethnicity and Z or HT^2^/Z was also examined (Figure 1ii). If present, such an interactive effect would imply that the difference in FFM between ethnic groups varies with different values of Z or HT^2^/Z, providing different slopes and different intercepts (Figure 1iii). Finally, models with similar R^2^, SEE and BIC may have very similar average residuals. However, the residuals might be unevenly distributed across the ethnic groups (Figure 1iv). To ensure that the ‘best’ model had an even distribution of residuals among ethnic groups (Figure 1v), we plotted residuals against HT^2^/Z and compared Pearson's correlation coefficient across models with similar R^2^, SEE and BIC.

## Results

In total, 1575 children (Mean (SD) age 8.1 (1.6) years; 47% boys) were included in the body size population and 698 (8.5 (1.7) years; 46% boys) children were included in the BIA calibration population, with 355 children participating in both parts of the study (Figure S1). These two populations encompassed a similar age range and distribution of ethnicities, though the BIA calibration population was slightly older, heavier and taller than the body size population (Table 1).

**Table 1:** Background characteristics of the study populations.

	*White*	*Black African/Caribbean*	*South Asian*	*Other*
**Body size population (n = 1575)**				
N (% boys)	574 (49%)	488 (41%)	311 (50%)	202 (47%)
Age, years	8.1 (1.6)	8.2 (1.6)	8.1 (1.6)	8.1 (1.7)
z-Height#	0.24 (1.0)	0.90 (1.0)	0.07 (1.1)	0.30 (1.1)
z-Weight#	0.38 (1.1)	0.98 (1.1)	0.00 (1.4)	0.45 (1.3)
z-Body mass index#	0.34 (1.1)	0.74 (1.3)	−0.07 (1.4)	0.42 (1.3)
**BIA calibration population (n = 698)**				
N (% boys)	242 (48%)	196 (45%)	148 (50%)	112(43%)
Age, years	8.5 (1.7)	8.5 (1.8)	8.5 (1.7)	8.6 (1.7)
z-Height#	0.33 (1.0)	1.00 (1.1)	0.35 (1.2)	0.43 (1.1)
z-Weight#	0.45 (1.1)	1.10 (1.2)	0.28 (1.5)	0.57 (1.2)
z-Body mass index#	0.37 (1.2)	0.92 (1.4)	0.14 (1.5)	0.48 (1.3)

Data presented as mean (SD) unless otherwise stated.

# Height, weight and body mass index were adjusted for age and sex and expressed as z (or SD score) using British 1990 reference [31].

### Comparison of body size and proportions between ethnic groups

**Figure 2:**
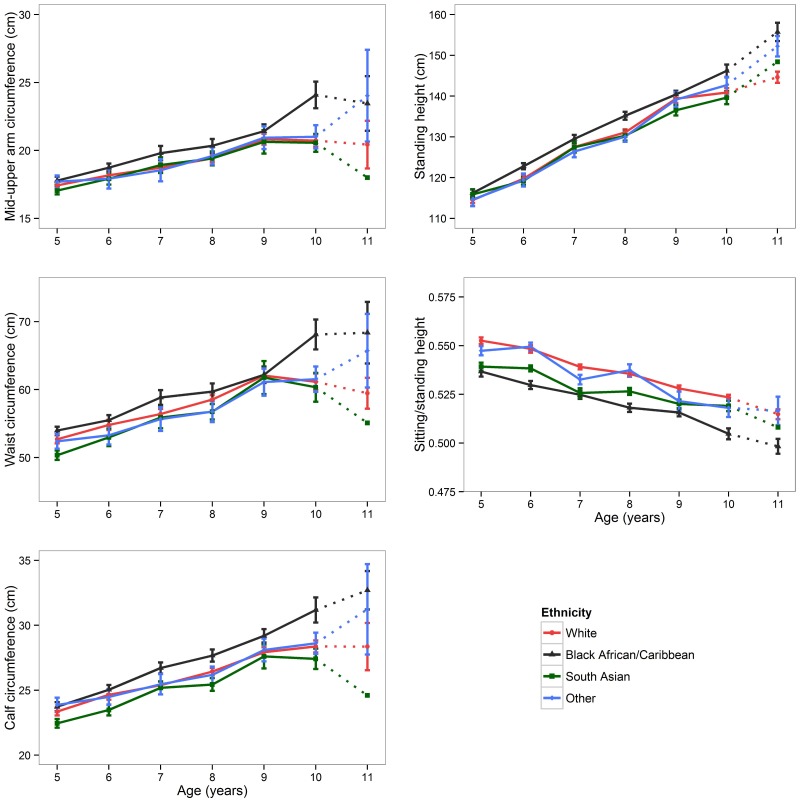
Ethnic differences in anthropometric measurements in boys. Dots represent mean values, error bars represent standard error of the mean. *Black African/Caribbean* boys generally had higher mean values of all circumferences and standing height, but lower sitting/standing height ratio, at each age group as compared to *White* and *South Asian* children. An increasing trend for ethnic differences in body circumferences is evident, especially after 9 years of age. Despite its heterogeneity, the ‘*Other*’ group tend to track *White* and *South Asian* group more closely than *Black African/Caribbean* group. Similar pattern was observed in girls (Figure S3). Due to relatively small sample size >10 years old (n = 12) the estimates may be biased therefore the trend lines from 10 to 11 years old were replaced by dotted lines.

There were marked ethnic differences in body size, these differences being similar in both populations (Table 1). After adjusting for age and sex, *Black African/Caribbean* children were significantly heavier, taller and with greater BMI than those from other ethnic groups. On average, *Black African/Caribbean* children had the largest body girths (MUAC, calf and waist) across all age groups, but the lowest sitting/standing height ratio when compared to other ethnicities, indicating relatively longer legs (Figure 2 and Figure S3). With the exception of height and sitting/standing height ratio, ethnic differences in body size increased after 9 years of age, particularly between *Black African/Caribbean* and *South Asian* children. The number of children in each age group is given in Table S1.

### Comparison of body composition between ethnic groups

**Figure 3:**
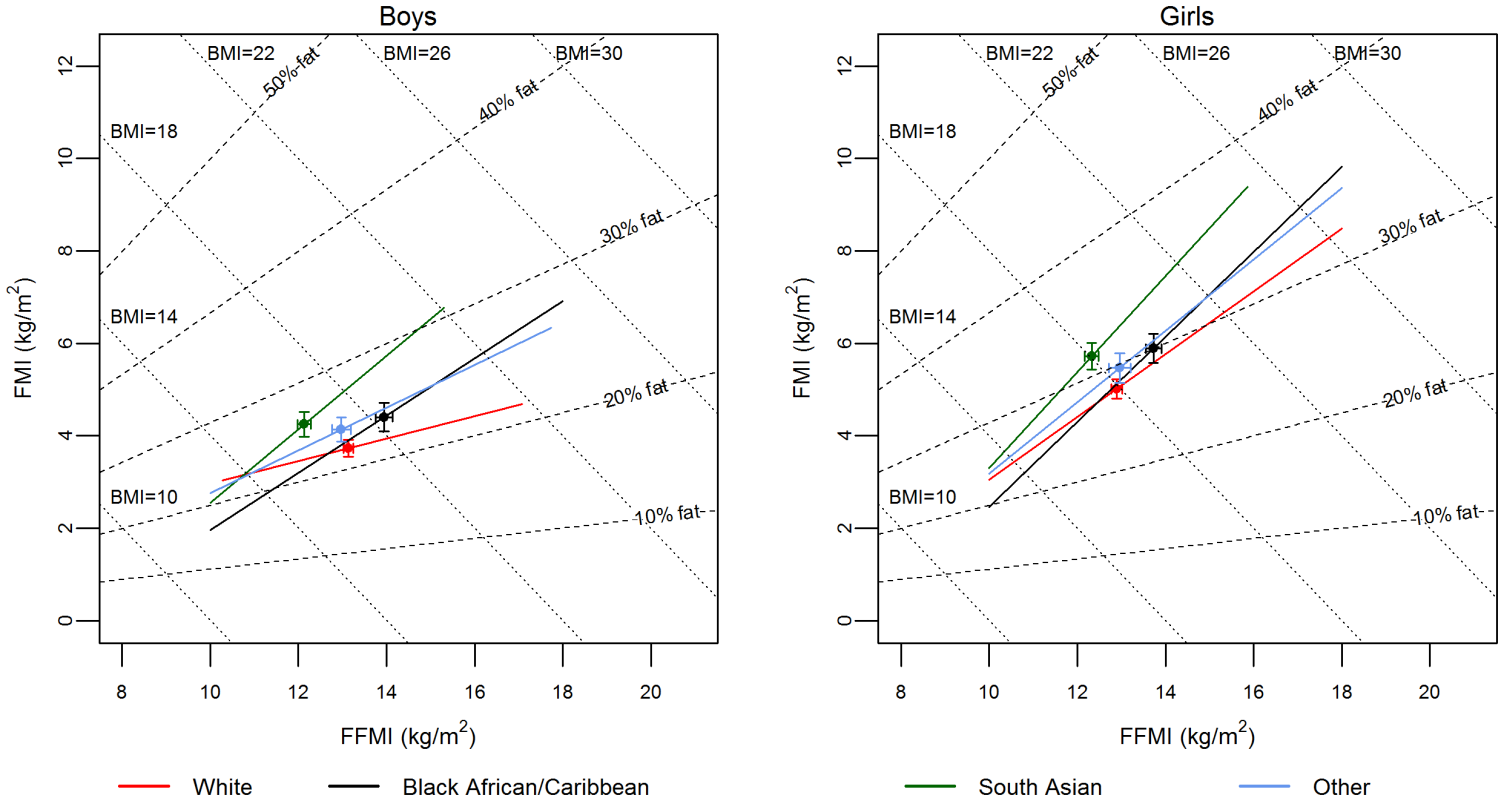
Hattori's body composition chart according to sex. Regression line for each ethnic group is shown with individual data points removed. Regression line is shown from fat-free mass index (FFMI) 10 to 18 due to fewer individuals at both ends of the regression line. Error bars show standard error of the mean. There were no significant ethnic differences in fat-mass index (FMI) for either boys or girls. In both boys and girls, FFMI was significantly higher in *Black African/Caribbean* children than in *White* (boys: p<0.001, girls: p<0.001), *South Asian* (boys: p<0.001, girls: p<0.001) and ‘*Other*’ (boys: p<0.01, girls: p<0.05) children.

FMI and FFMI for each child in the BIA calibration population were plotted on Hattori charts (Figure 3). For a particular BMI value, there was substantial individual variability in the proportions of FFMI and FMI (Figure S4). The standard deviation of FFMI for all children was 1.66 kg/m^2^ and of FMI was 2.62 kg/m^2^. This indicates that after adjusting for height, overall between-subject variability in FM was over 1.6 times greater than that in FFM. In contrast, after adjusting for height, ethnic differences in FFM were more evident than ethnic differences in FM (Figure 3). There were also clear ethnic differences in the distribution of FM and FFM, i.e. after adjusting for height, as FFM increased, the FM in *South Asian* and *Black African/Caribbean* children increased more than in *White* children, as demonstrated by steeper regression lines. FMI was generally higher in *South Asian* children than children of other ethnicities for both boys and girls. The ethnic-specific relationship between FMI and FFMI also differed in boys and girls, with a much steeper relationship in girls, indicating that after adjusting for height, girls had greater FM per unit of FFM than boys.

### Deriving BIA calibration equations

Two approaches were used to estimate FFM. In general, equations that contained HT^2^/Z (group A) fitted the data better, as shown by their diagnostic criteria, than equations where height and Z were fitted separately (group B). In particular, models in group A had higher R^2^, lower SEE and lower BIC as compared to those in group B (Table 2). In group A, although four models achieved the same R^2^ and SEE, model A2 was considered the ‘best’ as it had the lowest BIC. In group B, the ‘best’ equation was model B2, which was similar to model A2, except that height and Z were fitted separately. The coefficients (95% confidence intervals) for all ten models are shown in detail in Table S2.

**Table 2:** Models' selection criteria.

	Independent variables	R^2^	SEE	BIC; df
*Group A*				
Model A1	HT^2^/Z	0.92	1.85	2859; 3
Model A2	HT^2^/Z+Weight	0.94	1.61	2665; 4
Model A3	HT^2^/Z+Weight+Ethnicity	0.94	1.61	2684; 7
Model A4	HT^2^/Z+Weight+Ethnicity+Sex	0.94	1.61	2689; 8
Model A5	HT^2^/Z+Weight+Ethnicity+Sex+Ethnicity*HT^2^/Z	0.94	1.61	2705; 11
*Group B*				
Model B1	Height+Z	0.90	2.09	3034; 4
Model B2	Height+Z+Weight	0.93	1.73	2771; 5
Model B3	Height+Z+Weight+Ethnicity	0.93	1.72	2780; 8
Model B4	Height+Z+Weight+Ethnicity+Sex	0.93	1.71	2782; 9
Model B5	Height+Z+Weight+Ethnicity+Sex+Ethnicity*Z	0.93	1.69	2777; 12

Abbreviations: HT^2^/Z: height^2^/impedance; R^2^: proportion of variance explained from each model; SEE: Standard Error of the Estimate; BIC: Bayesian Information Criterion; df: degrees of freedom. Fat-free mass was the dependent variable which was regressed on all other variables as described in the table.

**Figure 4:**
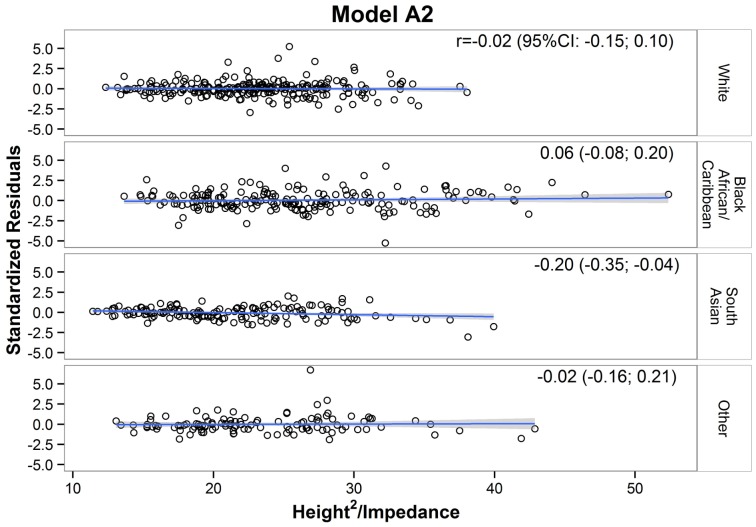
Residuals distribution for each ethnic group for model A2. Residuals from model A2 were regressed, for each ethnic group, on height^2^/impedance as indicated by the bold regression line. The shaded area represents a 95% confidence interval for the mean of residuals as a function of height^2^/impedance. Correlation coeffients (95% confidence interval) are also shown. No mean, and no slope, differed significantly from zero.

Out of all ten models, model A2, which had the lowest BIC, was considered the ‘best’. When plotting residuals (deuterium derived FFM – model estimated FFM) against HT^2^/Z for model A2 (Figure 4), the correlation coefficient in each ethnic group was low (i.e. r<|0.3|), suggesting that the equation did not have any significant ethnic-bias. Furthermore, none of the other models had residuals that were more evenly distributed than those from model A2 (Figure S5), confirming that the latter was the ‘best’ approach. The calibration equation derived from model A2 was as follows:
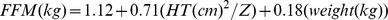



## Discussion

In this study, we examined whether there were ethnic differences in body size, proportions and composition in a multi-ethnic population of children, and whether the use of ethnic-specific calibration equations, which convert bioelectrical data into FFM, was required. We demonstrated that despite observing ethnic differences in body size, composition and proportions, an ethnic-specific calibration equation was not required for children between 5 and 11 years of age.

Our study had several strengths. The relatively large sample size allowed us to address the ethnic component of BIA calibration to a high standard, thus minimizing any ethnic bias. In the isotope dilution analysis, a clear record of how much fluid was consumed in the four hours after the administration of isotope drink was obtained from all children, therefore ensuring the estimation of FFM was accurate. Many primary school children were involved in exercise during school time, and may have consumed variable amounts of fluid.

However, the study is not without its limitations. The cross-sectional design of this study means that we could not compare the performance of the equations in the same children over time. Second, the ‘*Other*’ group was poorly defined and was likely to include a wide range of ethnicity, as well as those of mixed ethnicity. Further work will be required to address the full spectrum of ethnicity. However, results from this study remained very similar after excluding the ‘*Other*’ group, with the ‘best’ calibration equation still in the form of FFM = HT^2^/Z+weight, suggesting their inclusion did not bias results. Finally, since our study sample were all recruited from children attending primary schools, our calibration equation is unlikely to be suitable for a more diverse population, particularly in exclusively overweight populations [35], [36].

Two study populations were used in our investigation, with the BIA calibration population being slightly taller, heavier and older. Given that both populations were recruited in London, had similar ethnic profile and covered similar ranges of BMI and height (Figure S2), we are confident that both populations are representative of each other.

In previous studies, BIA equations were evaluated only according to their ability to explain variability in the outcome (R^2^) and the magnitude of predictive error in individuals (SEE). However, these indicators do not provide any information on whether the unexplained variability (residuals) is distributed evenly among the sub-groups. We have demonstrated here that by plotting residuals against the biggest predictor of FFM (HT^2^/Z), we could derive correlation coefficients and visually inspect the distribution of residuals. This approach ensures that the ‘best’ calibration equation chosen has minimal ethnic bias.

Some calibration studies have taken the approach of randomly separating the study population into two groups, one for generating the equation and one for validating the equation. However, this approach results in loss of statistical power, and potentially in reduced accuracy of the regression coefficients. We therefore elected to use the entire sample to generate the BIA equation.

BMI is a poor indicator of body fatness, especially in a multi-ethnic population. Indeed, consistent with earlier work in older children [9]–[11], [18], [19], our data, presented on the Hattori charts (Figure 3, Figure S4), showed firstly, that BMI can embrace a wide range of fatness, and secondly, that this range of fatness was ethnic-specific. We interpreted the Hattori charts with great caution as there were fewer children at either end of FFMI and FMI. However, even if observations were limited to the mid-range of FFMI and FMI where we had the most data, clear ethnic differences can be seen. For example, the same BMI value is more likely to represent a higher amount of fat in *South Asian* than in *White* or *Black African/Caribbean* children. This difference in range of fatness increased in older age groups, as suggested by the diverging regression lines and more widely spread data points on the Hattori chart. Indeed, ethnic differences in body composition have been demonstrated in populations with older children [11], adolescents [19] and adults [37].

BIA offers substantial benefits over BMI as it provides relative proportions of FM and FFM. It achieves this by sending a current through the body and measuring the impedance, which is the resistance to the flow of current. Since tissues containing less water impede the flow of current to a greater extent than tissues containing more water, impedance can be converted to FFM through the use of a calibration equation. The conventional BIA model assumes that the body has a cylindrical shape with constant cross-sectional area from top to bottom, leading to the wide-spread use of the impedance index (HT^2^/Z) in calibration equations. Consistent with this theory, our study found that this approach provided better estimates of FFM than fitting height and Z separately.

Previous calibration studies in the US, New Zealand and Mexico found no significant contribution of ethnicity to the calibration equation, despite ethnic differences in body composition found in the samples [38]–[40]. These studies had a different ethnic mix and included other ethnic classifications such as Maori and Pacific islanders, indigenous and non-indigenous Mexican. In addition, they covered a wider age range than ours, including elderly adults. As such, their equations are unlikely to be suitable for our population. Although our calibration equation is relatively similar to that derived from a previous study conducted in a multi-ethnic sample of children from New Zealand [38], the two equations generate body composition values in *White* children that may differ by 3% in FFM, equivalent to 15% in FM (Figure S6). This occurs because the same absolute error in FFM and FM is proportionally higher in FM, since the latter represents the smaller component of weight. This discrepancy reaffirms the importance of using a calibration equation derived from a population with similar characteristics to the population of interest.

Previous calibration studies in multi-ethnic populations in the UK at older ages have indicated that ethnic-specific equations may be required [18], [19]. Considering the conceptual illustration described previously (Figure 1), these calibration equations in multi-ethnic population are of type ii [19] and iii [18]. Our calibration equation is however not ethnic-specific (type i). This difference in findings could suggest that ethnic differences in FFM in our population were smaller than those in previous studies. However, comparison of the ethnic variability of FFM in our study with that from a similar study of older children [18] does not suggest that this is the case, since the percentage difference in mean FFM between *White* and *Black African/Caribbean* children and *White* and *South Asian* children are very similar (*White* vs *Black African/Caribbean*: Nightingale vs. Lee: 14.3% vs. 13.6%; *White* vs *South Asian*: −5.7% vs. −5.4% respectively).

The magnitude of ethnic differences in body size and proportion, in particular the limbs, are likely to contribute to contrasting findings between studies. Although BIA assumes the body is a cylinder with the same cross-sectional area, in reality the body is made up of several cylinders with different cross sectional areas and different lengths. When an electrical current passes through a limb which is comparatively longer and thinner, the impedance generated would be greater than when the current passes through the wider trunk. Indeed, it has been demonstrated that the lower limb can have a relative resistance over 8 times that of the trunk [41], therefore contributing more to whole-body impedance. Variability in the size or the length of the limbs is likely to have a bigger influence on impedance than similar variability in the trunk. In our study, there is some evidence to suggest that older children may display larger ethnic difference in limb size. The largest ethnic differences in MUAC and calf circumference were found at age 10 years, with possible further increases in older children. The observed increase in ethnic variability after 10 years of age was likely to be caused by different onsets of puberty, with *Black African/Caribbean* children entering puberty earlier than other ethnic groups [42]–[44]. This increase in ethnic variability is likely to persist after puberty and into adulthood.

The fact that the majority of our population were younger than those in previous studies which have reported ethnic-specific equations [18]–[20], suggests that ethnic differences in body size and proportions may be lower in young children. In addition, variability in ethnic composition of the study populations may have contributed to contrasting study findings. It is known that even within an ethnic group (e.g. *South Asian*), there are substantial differences in anthropometric measurements within each ethnic sub-groups (e.g., Indian, Pakistani, Bangladeshi) [45]. Such differences in ethnic profile may contribute to differences between calibration equations.

## Conclusion

We have shown that although ethnic differences in body size, proportion and composition are evident, these differences do not appear to impact on the relationship between HT^2^/Z and FFM, thus ethnic-specific terms are not required in the BIA calibration equation for this age group.

## Supporting Information

Figure S1 Flow chart of the two study populations i) body size population and ii) BIA calibration population.
(TIFF)

Figure S2 Distribution of height and body mass index z-scores in the body size and BIA calibration population. The ranges of height and BMI z-scores were similar in the body size population and BIA calibration population, suggesting the BIA calibration population is representative of the body size population. The BMI histogram in the BIA calibration population does however have a lower “peak” compared to the body size population, demonstrating that we successfully oversampled children who were further away from the mean, ensuring as wide a distribution of BMI as possible within each ethnic group.
(TIF)

Figure S3 Ethnic differences in anthropometric measurements in girls. Dots represent mean values, bars represent standard error of the mean. *Black African/Caribbean* girls had in general higher mean values of all circumferences and standing height, but lower sitting/standing height ratio, at each age group when compared to *White* and *South Asian* children. Despite its heterogeneity, the ‘*Other*’ group tended to track *White* and *South Asian* group better than *Black African/Caribbean* group. Due to relatively small sample size >10 years old (n = 12) the estimates may be biased therefore the trend lines from 10 to 11 years old were replaced by dotted lines. An earlier entry into puberty by the *Black African/Caribbean* girls may contribute to their contrast with the other groups over the age range. The findings for boys were similar (Figure 2).
(TIF)

Figure S4 Hattori's body composition chart according to sex. Fat-free mass (FFM) was estimated by deuterium dilution analysis in children in the BIA calibration population. Fat mass (FM) was calculated by subtracting FFM from weight. FFM index (FFMI) and FM index (FMI) were calculated by dividing FFM and FM by height (m^2^). Using the colour codes given at the foot of the graph, each point on the graph represents one child and each line represents the regression line for that ethnic group. For a particular BMI, there can be a wide variability of FFM and FM of children with the same height, while the average association also differs systematically between ethnic groups.
(TIF)

Figure S5 Relationship between residuals derived from models A2, A3, A4 and A5 and height^2^/impedance, by ethnic group. No trend was observed between standardized residuals and the height^2^/impedance term in each ethnic group in any of the models tested, since the magnitude of the correlation was found to be low (i.e. r<|0.3|).
(TIF)

Figure S6 Comparison of our equation and Rush's et al (2003) equation in *White* children. Our equation: Fat-free mass (FFM) = 1.12+0.71*(height^2^/impedance)+0.18*Weight; R2 = 0.94, SEE = 1.61 kg; Rush (2003) equation: FFM = 1.17+0.62*(height^2^/impedance)+0.23*Weight; R2 = 0.96, SEE = 2.44 kg. Despite the apparent similarity in the equations between our study and that by Rush et al, these calibration equations are still population-specific. Applying Rush's equation to *White* children in our BIA calibration population is likely to underestimate their FFM. For example, in a 10-year-old *White* child with low BMI, FFM would be underestimated by approximately 0.75 kg on average, which is equivalent to around 3% of FFM in the child. In a two-component model in which body weight is divided into fat mass (FM) and FFM, this underestimation would also mean an overestimation of FM by 0.75 kg, which is equivalent to around 15% of FM in the child.
(TIF)

Table S1 Distribution of boys and girls with measurements on body size and proportions by age group and ethnicity.
(DOCX)

Table S2 Regression coefficients (95% confidence intervals) from modelling fat-free mass by fitting height^2^/impedance (models of Group A) or by fitting height and impedance separately (models of Group B).
(DOCX)

Text S1 Additional information on anthropometric measurements.
(DOCX)
