# Supplementary figures and images for: Ethnic Variability in Body Size, Proportions and Composition in Children Aged 5 to 11 Years: Is Ethnic-Specific Calibration of Bioelectrical Impedance Required?
Source: PLoS One. 2014 Dec 5;9(12):e113883. doi: 10.1371/journal.pone.0113883 (PMC4257615; doi:10.1371/journal.pone.0113883)

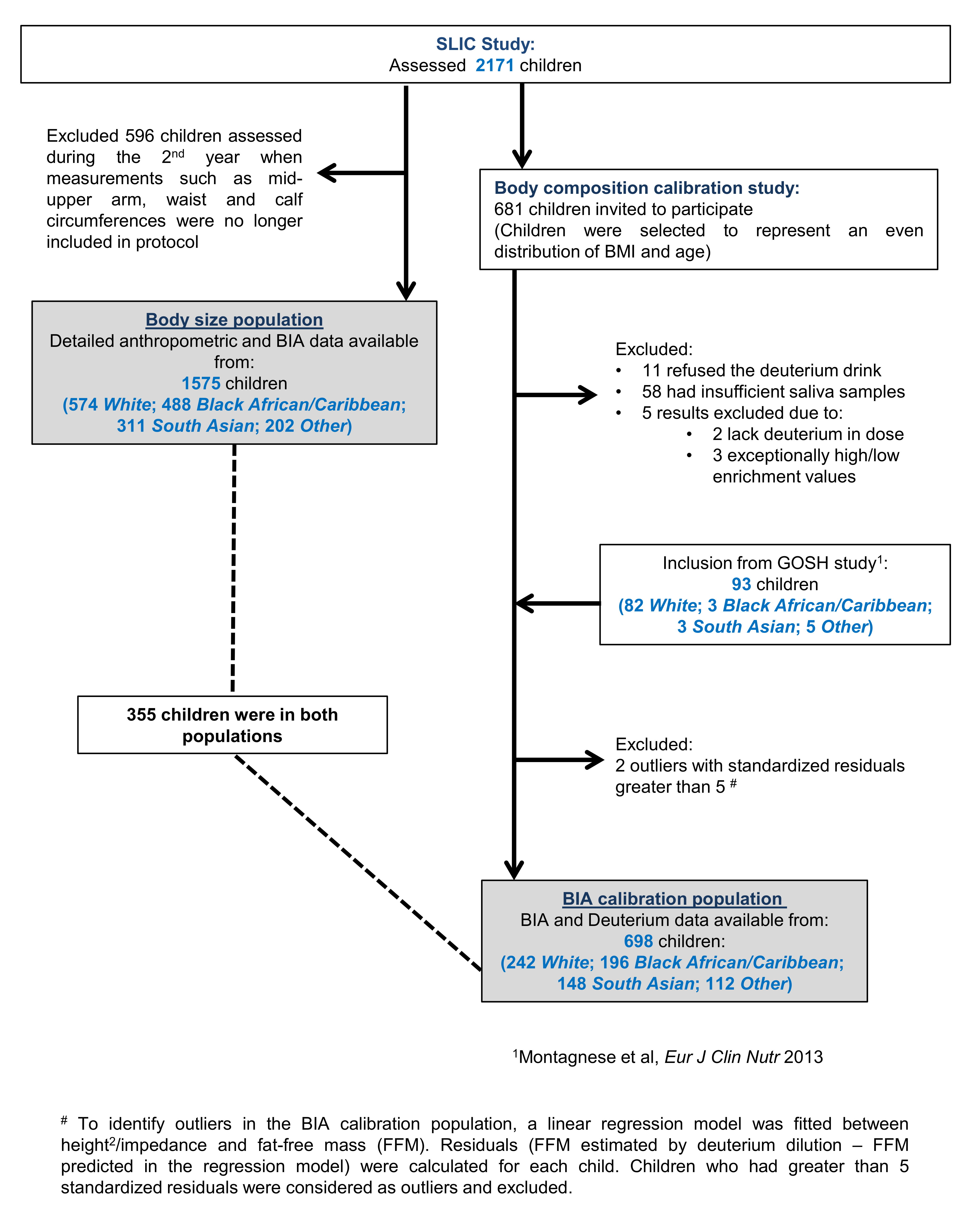

Supplement: Figure S1 — Flow chart of the two study populations i) body size population and ii) BIA calibration population. (TIFF) [file pone.0113883.s001.tiff]

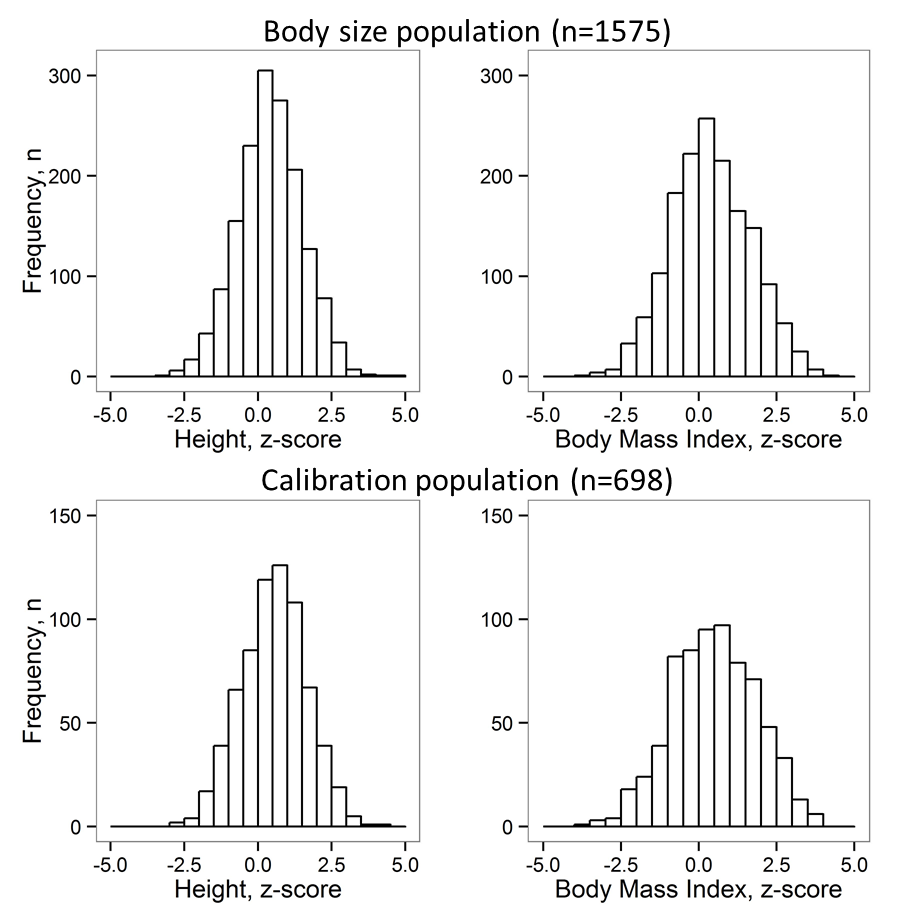

Supplement: Figure S2 — Distribution of height and body mass index z-scores in the body size and BIA calibration population. The ranges of height and BMI z-scores were similar in the body size population and BIA calibration population, suggesting the BIA calibration population is representative of the body size population. The BMI histogram in the BIA calibration population does however have a lower “peak” compared to the body size population, demonstrating that we successfully oversampled children who were further away from the mean, ensuring as wide a distribution of BMI as possible within each ethnic group. (TIF) [file pone.0113883.s002.tif]

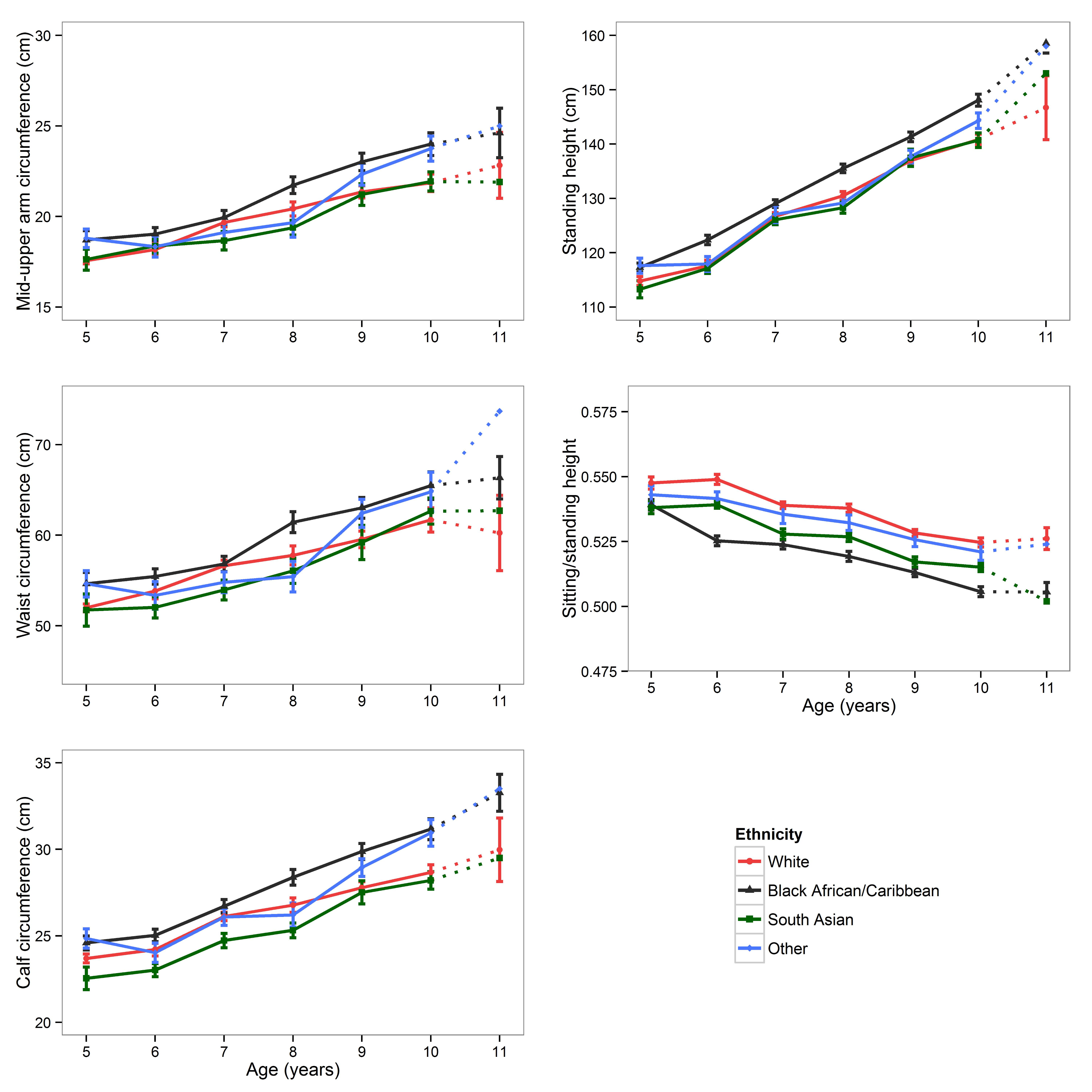

Supplement: Figure S3 — Ethnic differences in anthropometric measurements in girls. Dots represent mean values, bars represent standard error of the mean. Black African/Caribbean girls had in general higher mean values of all circumferences and standing height, but lower sitting/standing height ratio, at each age group when compared to White and South Asian children. Despite its heterogeneity, the ‘Other’ group tended to track White and South Asian group better than Black African/Caribbean group. Due to relatively small sample size >10 years old (n = 12) the estimates may be biased therefore the trend lines from 10 to 11 years old were replaced by dotted lines. An earlier entry into puberty by the Black African/Caribbean girls may contribute to their contrast with the other groups over the age range. The findings for boys were similar (Figure 2). (TIF) [file pone.0113883.s003.tif]

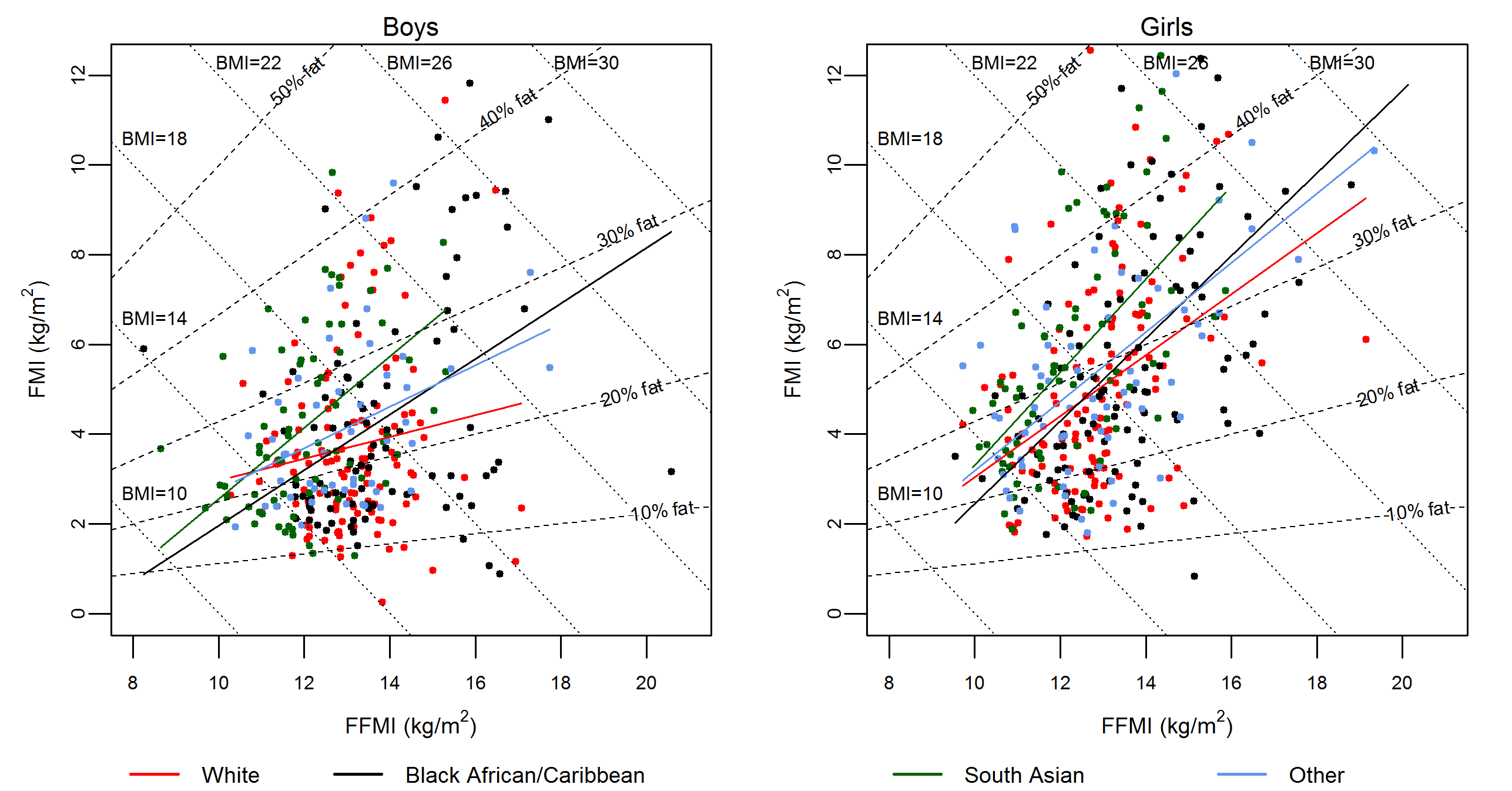

Supplement: Figure S4 — Hattori's body composition chart according to sex. Fat-free mass (FFM) was estimated by deuterium dilution analysis in children in the BIA calibration population. Fat mass (FM) was calculated by subtracting FFM from weight. FFM index (FFMI) and FM index (FMI) were calculated by dividing FFM and FM by height (m2). Using the colour codes given at the foot of the graph, each point on the graph represents one child and each line represents the regression line for that ethnic group. For a particular BMI, there can be a wide variability of FFM and FM of children with the same height, while the average association also differs systematically between ethnic groups. (TIF) [file pone.0113883.s004.tif]

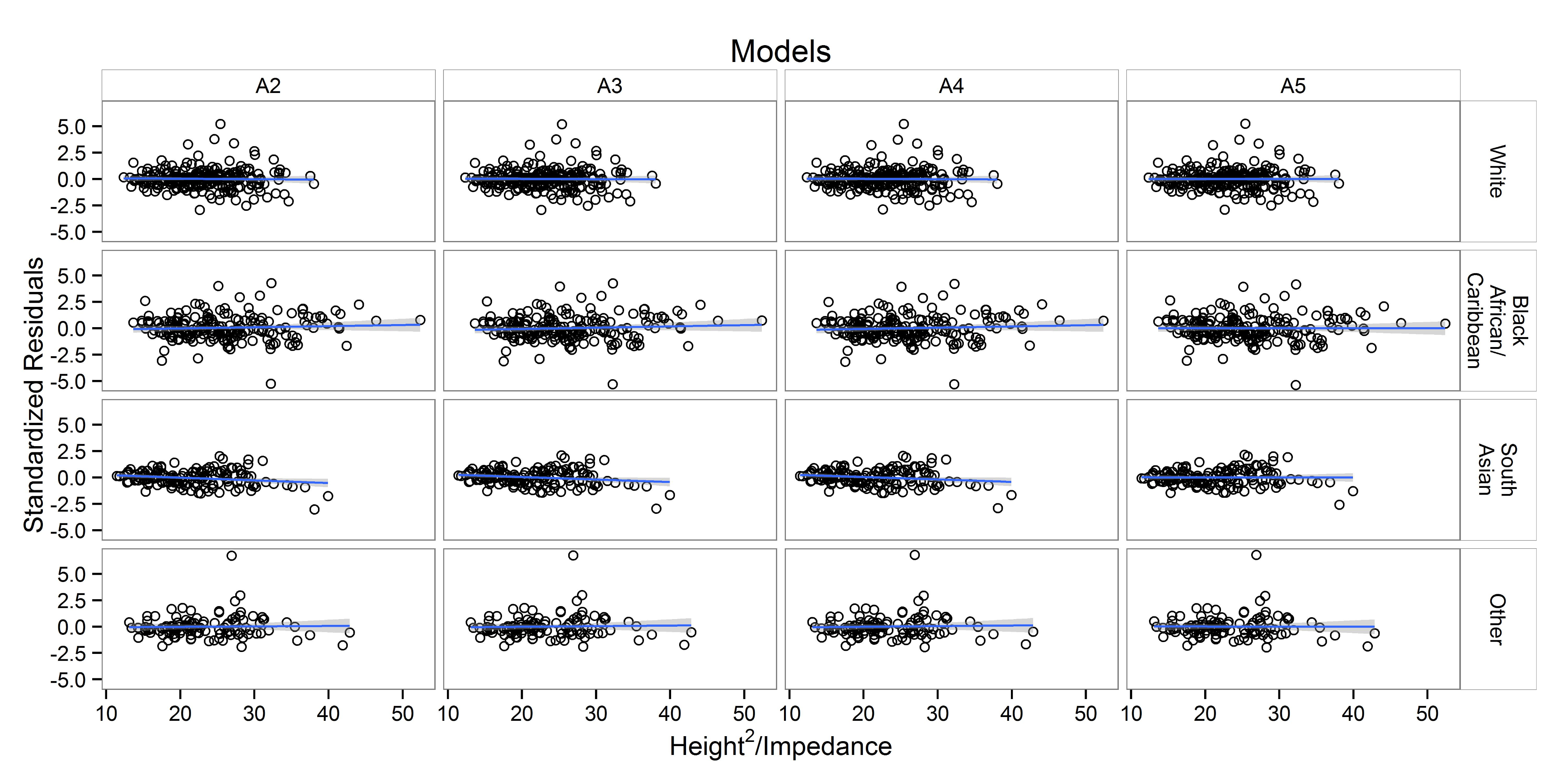

Supplement: Figure S5 — Relationship between residuals derived from models A2, A3, A4 and A5 and height2/impedance, by ethnic group. No trend was observed between standardized residuals and the height2/impedance term in each ethnic group in any of the models tested, since the magnitude of the correlation was found to be low (i.e. r<|0.3|). (TIF) [file pone.0113883.s005.tif]

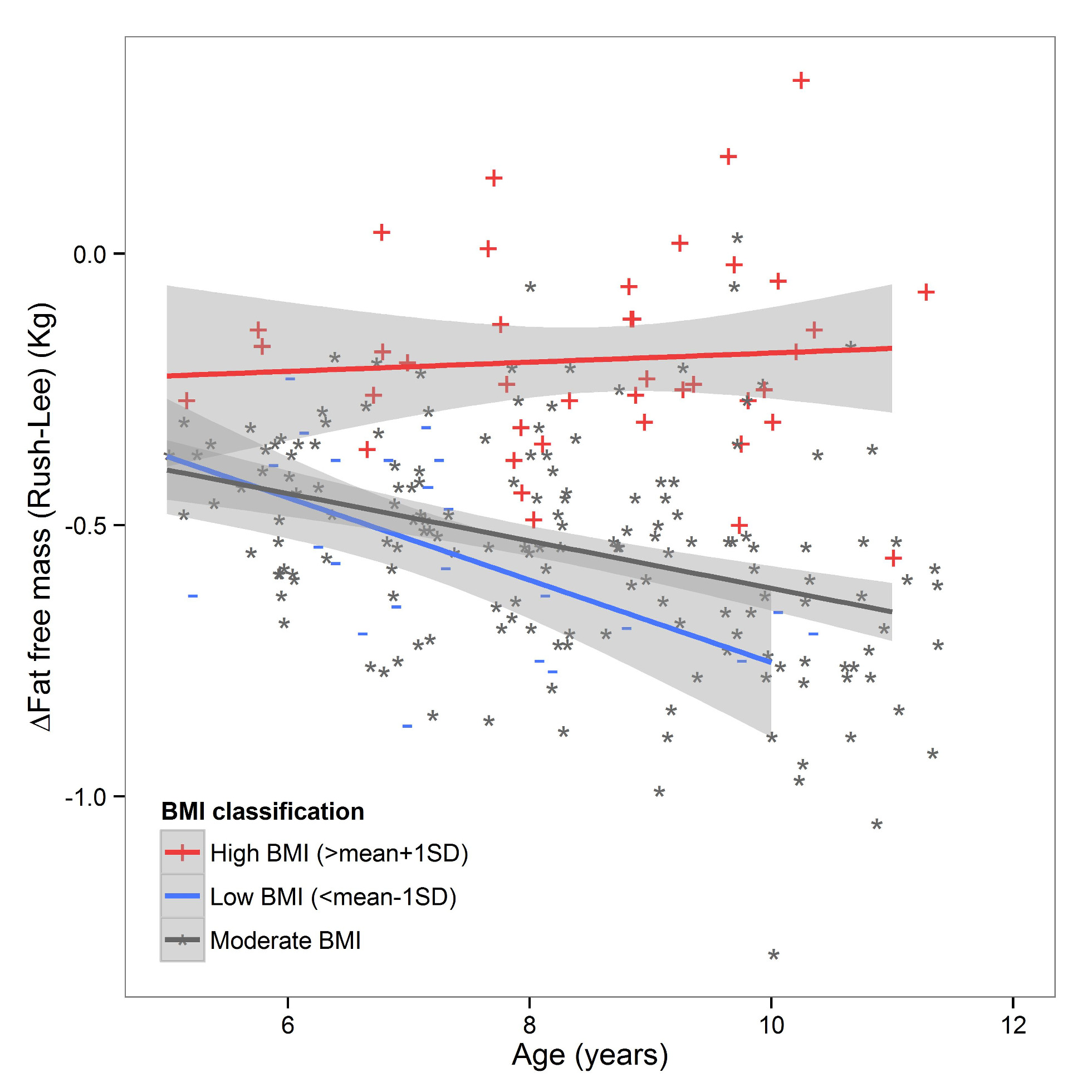

Supplement: Figure S6 — Comparison of our equation and Rush's et al (2003) equation in White children. Our equation: Fat-free mass (FFM) = 1.12+0.71*(height2/impedance)+0.18*Weight; R2 = 0.94, SEE = 1.61 kg; Rush (2003) equation: FFM = 1.17+0.62*(height2/impedance)+0.23*Weight; R2 = 0.96, SEE = 2.44 kg. Despite the apparent similarity in the equations between our study and that by Rush et al, these calibration equations are still population-specific. Applying Rush's equation to White children in our BIA calibration population is likely to underestimate their FFM. For example, in a 10-year-old White child with low BMI, FFM would be underestimated by approximately 0.75 kg on average, which is equivalent to around 3% of FFM in the child. In a two-component model in which body weight is divided into fat mass (FM) and FFM, this underestimation would also mean an overestimation of FM by 0.75 kg, which is equivalent to around 15% of FM in the child. (TIF) [file pone.0113883.s006.tif]
