# Supplementary material for: Ethnic Variability in Body Size, Proportions and Composition in Children Aged 5 to 11 Years: Is Ethnic-Specific Calibration of Bioelectrical Impedance Required?
Source: PLoS One. 2014 Dec 5;9(12):e113883. doi: 10.1371/journal.pone.0113883 (PMC4257615; doi:10.1371/journal.pone.0113883)
Supplement: Table S1 — Distribution of boys and girls with measurements on body size and proportions by age group and ethnicity. (DOCX) [file pone.0113883.s007.docx]

**Table S1 Distribution of boys and girls with measurements on body size and proportions by age group and ethnicity**

|  | ***White*** | ***Black African/ Caribbean*** | ***South Asian*** | ***Other*** |
| --- | --- | --- | --- | --- |
| ***Boys, n*** |  |  |  |  |
| 5 to <6 years | 40 | 31 | 22 | 16 |
| 6 to <7 years | 36 | 28 | 31 | 15 |
| 7 to <8 years | 62 | 38 | 27 | 17 |
| 8 to <9 years | 56 | 37 | 32 | 18 |
| 9 to <10 years | 48 | 43 | 19 | 15 |
| 10 to <11 years | 36 | 19 | 23 | 11 |
| 11 to <12 years | 3 | 5 | 1 | 3 |
| ***Girls, n*** |  |  |  |  |
| 5 to <6 years | 39 | 27 | 15 | 12 |
| 6 to <7 years | 33 | 49 | 31 | 19 |
| 7 to <8 years | 69 | 52 | 17 | 20 |
| 8 to <9 years | 40 | 47 | 33 | 13 |
| 9 to <10 years | 64 | 63 | 21 | 25 |
| 10 to <11 years | 44 | 43 | 38 | 17 |
| 11 to <12 years | 4 | 6 | 1 | 1 |
