# Supplementary material for: Ethnic Variability in Body Size, Proportions and Composition in Children Aged 5 to 11 Years: Is Ethnic-Specific Calibration of Bioelectrical Impedance Required?
Source: PLoS One. 2014 Dec 5;9(12):e113883. doi: 10.1371/journal.pone.0113883 (PMC4257615; doi:10.1371/journal.pone.0113883)
Supplement: Table S2 — Regression coefficients (95% confidence intervals) from modelling fat-free mass by fitting height2/impedance (models of Group A) or by fitting height and impedance separately (models of Group B). (DOCX) [file pone.0113883.s008.docx]

| **Group A models** |  |  |  |  |  |
| --- | --- | --- | --- | --- | --- |
|  | Model | | | | |
|  | A1 | A2 | A3 | A4 | A5 |
|  | b (95% CI) | b (95% CI) | b (95% CI) | b (95% CI) | b (95% CI) |
| Constant | 0.18 (-0.37; 0.72) | 1.12 (0.64; 1.61) | 1.21 (0.68; 1.74) | 1.16 (0.62; 1.69) | 1.26 (0.32; 2.21) |
| HT^2^/Z (per unit) | 1.00 (0.98; 1.02) | 0.71 (0.67; 0.76) | 0.71 (0.67; 0.75) | 0.70 (0.66; 0.75) | 0.70 (0.65; 0.76) |
| Weight (per kg) |  | 0.18 (0.15; 0.20) | 0.18 (0.15; 0.20) | 0.18 (0.16; 0.21) | 0.18 (0.16; 0.20) |
| Ethnicity (baseline: *White*) |  |  |  |  |  |
| *Black African/Caribbean* |  |  | 0.06 (-0.25; 0.37) | 0.06 (-0.25; 0.37) | -0.54 (-1.82; 0.75) |
| *South Asian* |  |  | -0.13 (-0.46; 0.21) | -0.14 (-0.48; 0.20) | 0.54 (-0.82; 1.91) |
| *Other* |  |  | -0.04 (-0.40; 0.33) | -0.03 (-0.39; 0.33) | -0.37 (-1.91; 1.17) |
| Male (baseline: Females) |  |  |  | 0.15 (-0.10; 0.40) | 0.14 (-0.12; 0.39) |
| Ethnicity*Ht^2^/Z (baseline: *White*) |  |  |  |  |  |
| *Black African/Caribbean *HT^2^/Z* |  |  |  |  | 0.02 (-0.03; 0.08) |
| *South Asian*Ht^2^/Z* |  |  |  |  | -0.03 (-0.09; 0.03) |
| *Other*Ht^2^/Z* |  |  |  |  | 0.01 (-0.05; 0.08) |

Table S2 Regression coefficients (95% confidence intervals) from modelling fat-free mass by fitting height^2^/impedance (models of Group A) or by fitting height and impedance separately (models of Group B)

| **Group B models** |  |  |  |  |  |
| --- | --- | --- | --- | --- | --- |
|  | Model | | | | |
|  | B1 | B2 | B3 | B4 | B5 |
|  | b (95% CI) | b (95% CI) | b (95% CI) | b (95% CI) | b (95% CI) |
| Constant | -11 (-14; -8.67) | -5.28 (-7.54; -3.03) | -6.01 (-8.30; -3.72) | -6.42 (-8.73; -4.10) | -8.52 (-12; -5.50) |
| Height (per cm) | 0.40 (0.38; 0.41) | 0.26 (0.24; 0.28) | 0.26 (0.24; 0.28) | 0.25 (0.23; 0.27) | 0.26 (0.24; 0.28) |
| Z (per Ω) | -0.02 (-0.02; -0.02) | -0.02 (-0.02; -0.01) | -0.01 (-0.02; -0.01) | -0.01 (-0.02; -0.01) | -0.01 (-0.02; -0.01) |
| Weight (per kg) |  | 0.21 (0.19; 0.24) | 0.21 (0.19; 0.24) | 0.22 (0.20; 0.25) | 0.21 (0.19; 0.24) |
| Ethnicity (baseline: *White*) |  |  |  |  |  |
| *Black African/Caribbean* |  |  | 0.38 (0.05; 0.71) | 0.38 (0.05; 0.71) | 5.01 (2.08; 7.94) |
| *South Asian* |  |  | -0.26 (-0.63; 0.11) | -0.30 (-0.67; 0.07) | -2.25 (-5.41; 0.91) |
| *Other* |  |  | 0.01 (-0.37; 0.40) | 0.02 (-0.37; 0.40) | 4.22 (0.91; 7.52) |
| Male (baseline: Females) |  |  |  | 0.29 (0.02; 0.56) | 0.26 (-0.01; 0.52) |
| Ethnicity*Z (baseline: *White*) |  |  |  |  |  |
| *Black African/Caribbean *Z* |  |  |  |  | -0.01 (-0.01; -0.002) |
| *South Asian*Z* |  |  |  |  | 0.002 (-0.002; 0.006) |
| *Other*Z* |  |  |  |  | -0.005 (-0.01; -0.001) |

**Abbreviations:** CI: Confidence interval, HT^2^/Z: height^2^/impedance; Z: impedance
