# Supplementary material for: Ethnic Variability in Body Size, Proportions and Composition in Children Aged 5 to 11 Years: Is Ethnic-Specific Calibration of Bioelectrical Impedance Required?
Source: PLoS One. 2014 Dec 5;9(12):e113883. doi: 10.1371/journal.pone.0113883 (PMC4257615; doi:10.1371/journal.pone.0113883)
Supplement: Text S1 — Additional information on anthropometric measurements. (DOCX) [file pone.0113883.s009.docx]

**Text S1 Additional information on anthropometric measurements**

Standing height was measured to the nearest 0.1 cm with no shoes using a portable Leicester stadiometer; sitting height, measured while sitting on a stool, was reported as child’s height minus stool height (60cm); weight was measured to the nearest 0.1 kg without shoes using Seca digital scales, circumference measurements were performed with Seca tape measure while ensuring the tape is in contact with skin but without compressing it.
